# Physicochemical Properties of Honey from Contract Beekeepers, Street Vendors and Branded Honey in Sabah, Malaysia

**DOI:** 10.21315/tlsr2022.33.3.5

**Published:** 2022-09-30

**Authors:** A H Robin Lim, Lum Mok Sam, Januarius Gobilik, Kimberly Ador, Jamilah Lee Nyuk Choon, Jonal Majampan, Suzan Benedick

**Affiliations:** 1 Faculty of Sustainable Agriculture, Universiti Malaysia Sabah, Locked Bag No.3, P.O. Box No. 3, 90509 Sandakan, Sabah, Malaysia; 2 Koperasi Pembangunan Desa, Wisma Pertanian Sabah, Jalan Tasik, Luyang, 88999, Kota Kinabalu, Sabah, Malaysia

**Keywords:** Honey, Physicochemical Properties, Mineral Content, Contract Beekeepers, Unknown Source, Branded Honey, Madu, Ciri-Ciri Fisikokimia, Kandungan Mineral, Penternak Lebah Kontrak, Sumber Asing, Madu Berjenama

## Abstract

The chemical properties of honey depend on the source of collection to packaging, but little is known about honey in Sabah. The aim of this study was to distinguish between the physicochemical properties and mineral content of 76 honey samples from local sources and supermarkets in Sabah, which were from contract beekeepers, unknown sources and branded honey. Raw honey was collected from contract beekeepers, while honey from unknown source was obtained from street vendors and wet markets, while branded honey was purchased from local supermarkets. The chemical parameters of the honey were assessed using established methods, while the mineral content of the honey was determined using inductively coupled plasma optical emission spectroscopy (ICP-OES). Significant differences were found in several parameters measured in honey from different sources, with principal component analysis (PCA) showing clear separation between the measured parameters, yielding five factors that accounted for up to 72.25% of the total explained variance. Honey from contract beekeepers showed significant differences and higher mineral content (Ca, Cu, Fe, K, Mg, Na and Zn) compared to honey from unknown source and branded honey. Potassium was the most important element in the study with an average of 2.65 g/kg and 629.4 mg/kg for sources from contract beekeepers and branded honey, respectively. The honey from the contract beekeepers was of better quality due to its high mineral content. The results suggest that honey from contract beekeepers could be a good choice when it comes to high mineral content.

## Abstract (fr)

Sifat kimia madu adalah bergantung kepada sumber pengumpulan hingga ke peringkat pembungkusan, namun demikian, maklumat tentang madu di Sabah kurang diketahui. Matlamat kajian ini adalah untuk membezakan antara sifat fisikokimia dan kandungan mineral bagi 76 sampel madu daripada sumber tempatan dan pasar raya di Sabah, iaitu daripada penternak lebah kontrak, sumber yang tidak diketahui dan madu berjenama. Madu mentah diperoleh daripada penternak lebah kontrak, manakala madu daripada sumber yang tidak diketahui telah diperoleh daripada peniaga jalanan dan pasar basah, manakala madu berjenama dibeli dari pasar raya tempatan. Parameter kimia madu dinilai menggunakan kaedah sedia ada yang telah ditetapkan, manakala kandungan mineral madu ditentukan menggunakan *inductively coupled plasma optical emission spectroscopy* (ICP-OES). Terdapat perbezaan bererti bagi beberapa parameter yang diukur dalam madu daripada sumber yang berbeza dan analisis komponen utama (PCA) menunjukkan perbezaan yang jelas antara parameter yang diukur, menghasilkan lima faktor yang menyumbang sehingga 72.25% daripada jumlah varians yang dijelaskan. Madu dari penternak lebah kontrak menunjukkan perbezaan yang ketara dan kandungan mineral yang lebih tinggi (Ca, Cu, Fe, K, Mg, Na dan Zn) berbanding madu dari sumber yang tidak diketahui dan madu berjenama. Kalium merupakan unsur terpenting dalam kajian dengan purata 2.65 g/kg dan 629.4 mg/kg masing-masing untuk sumber daripada penternak lebah kontrak dan madu berjenama. Madu dari penternak lebah kontrak adalah lebih berkualiti kerana kandungan mineralnya yang tinggi. Keputusan menunjukkan bahawa madu daripada penternak lebah kontrak boleh menjadi pilihan yang baik kerana ia mempunyai kandungan mineral yang tinggi.

**Highlights**
- Physicochemical properties and mineral content of 76 honey samples from contract beekeepers, unknown sources and branded honey in Sabah were tested.
- Significant differences were found in several parameters measured in honey from different sources.
- Honey produced from contract beekeepers were better in quality if mineral content is concerned.

## INTRODUCTION

Honey is defined by Codex Alimentarius (FAO & WHO 2019) as a “natural sweet substance produced by honey bees from the nectar of plants or secretions of living parts of plants or excretions of plant-sucking insects on the living parts of plants”. Since ancient times, honey bee-based products (honey, propolis and pollen) have been described as products that benefit the user. It has distinctive aromatic and organoleptic properties, that delight the consumer, and also carbohydrates, proteins, minerals and vitamins (Bogdanov *et al*. 2008; Solayman *et al*. 2016; Pita-Calvo & Vázquez 2017). Honey is used in both traditional and modern medicine (Boukraâ 2013). The best known are its antimicrobial properties to fight microbial infections, its anti-inflammatory properties to reduce swelling or inflammation, and its antioxidant properties to fight free radicals found in the human body (Idris *et al*. 2011; Ahmed & Othman 2013; Ahmed *et al*. 2018). Honey also contains secondary metabolites (e.g., phenolic compounds, compounds with nitrogenous base and terpenes) that can potentially be used in making drugs, flavours and fragrances (da Silva *et al*. 2016; Patrignani *et al*. 2018). In addition, honey bee-derived products have also been reported to increase livestock production, such as broiler chicken production for the meat industry (Attia *et al*. 2014; Zafarnejad *et al*. 2017). Due to its various benefits for consumers, honey is considered a superfood and its demand has steadily increased over the years.

However, purchasing preferences differ from one region to another. Arvanitoyannis and Krystallis (2005) identified quality, dietary, ethical and medicinal properties associated with honey as part of the motivation driving consumers to purchase in Romania; Batt and Liu (2012) added that brand, origin and cost were the main factors influencing consumer purchasing decisions in Perth, Australia. In Croatia, local producers were preferred, and medicinal and health benefits were cited as the main purchasing factors (Brščić *et al*. 2017). Buying honey from known sources is critical to ensure quality, authenticity, food safety and nutritional content; without clarity on sources, it will be difficult to identify adulterated or fake honey (Wu *et al*. 2017). It is also important to know that the quality, rheology and composition of honey can be affected by the processes during production (Elamine *et al*. 2020).

The production and marketing of honey from the western honey bee (*Apis mellifera*) accounts for the largest market share compared to that of the eastern honey bee (*A. cerana*), given its wide distribution around the world. In Sabah, Malaysia, honey production is mainly supported by *A. cerana* due to the populations found in the forests (Koeniger *et al*. 2010). The rearing location of the honey bee is important as the environment can influence the nutritional content of the honey produced – a particular environment can favour the production of high-quality honey that can be sold at a higher price (McDonald *et al*. 2018). Raising honey bees near orchards results in the production of honey with a flavour similar to that of the fruit, while a rearing site near agricultural plantations may expose the honey produced to pesticide residues (Connolly 2017). Contract beekeepers are registered beekeepers who have formed an agreement with the Sabah Rural Development Corporation (RDC) for the sustainable rearing and management of honey bees. This agreement includes that no pesticides are used in the immediate vicinity of the rearing and keeping of the honey bees and that the bees are not fed with sugar cane syrup or other substances than the natural nectar of the plants in the forest. Contract beekeepers raise honey bees in the forest, most of which produce multifloral or honeydew honey. The increasing demand for honey and concern for its quality have led to this study determining the physicochemical properties and mineral content of honey from contract beekeepers in Sabah and comparing it with honey from unknown sources and established brands.

## MATERIALS AND METHODS

### Chemicals and Reagents

Ultra-pure water was produced at a resistivity of >18.2 MΩ*cm by ELGA PURELAB Ultra, ELGA Labwater (Germany). Ethanol, isopropanol, 3-amino-5-nitrosalicylic acid, formic acid and nitric acid were purchased from Merck (Darmstadt, Germany). Gallic acid, 3-amino-5-nitrosalicylic acid, proline, ninhydrin, bovine serum albumin (BSA) and Folin-Ciocalteu reagent were purchased from Sigma-Aldrich, USA). Glucose and sodium carbonate were purchased from Classic Chemicals Sdn. Bhd. (Malaysia), and sodium bisulphite was purchased from R&M Chemicals (UK). All chemicals used were of analytical grade.

### Sample Collection

Raw honey samples were collected from various sources in Sabah with the help of beekeepers and officers from the RDC. Honey samples from RDC contract beekeepers (CB) were collected locally from July 2017 to July 2018, while honey from unknown sources (UNS) was purchased from street vendors (BH) along roads in the northern region of Sabah. To avoid duplication of samples, a survey was first conducted on branded honey (BH) purchased from local supermarkets in Sabah State, Malaysia from July 2017 to November 2018. Contract beekeepers raise honey bees (*A. cerana*) in the forest, about 15 to 60 min walk from their residential area. Honey samples were taken directly from the hive and sieved before being stored in a climate-controlled dark room (24°C). All honey collections were done in triplicate for quality assurance of the data.

### pH

The honey sample was diluted with ultra-pure water to a concentration of 10% (w/v) (Moniruzzaman *et al*. 2013). The pH was then determined using a pH meter (Mettler Toledo Seven Easy).

### Electrical Conductivity (EC)

Honey sample was diluted with ultra-pure water to a concentration of 20% (w/v) (Moniruzzaman *et al*. 2013). The EC of honey was determined using the EC meter (CyberScan Series 600, Eutech Instruments, Singapore).

### Colour (Pfund)

Honey samples of 50% (w/v) were prepared and heated up to 40°C. The colour was determined using spectrophotometer GENESYS™ 10S UV-Vis (Thermo Scientific, USA) at 635 nm following the method by White (1984):


(1) $$\text{Pfund}=-38.70+(371.39^{*}\text{Abs})$$

where Abs is absorbance reading.

### Colour Intensity (mAU)

Spectrophotometric method was used to measure the colour intensity of honey (Beretta *et al*. 2005). Honey was diluted with ultra-pure water to a concentration of 50% w/v and filtered with a 0.45 μm filter. The difference in absorbance reading at 450 nm and 720 nm was calculated and recorded as milli-Absorbance Units (mAU).

### Total Sugar Content (TSC)

Honey was placed on the refractometer (ATAGO Co., Ltd, Japan) and the reading was recorded. Ultra-pure water was used to standardise the refractometer to zero.

### Hydroxymethylfurfural (HMF) Content

Five grams of honey were weighed and dissolved in approximately 25 mL of distilled water. Then, 0.5 mL of Carrez solution I was added and mixed evenly. Next, 0.5 mL of Carrez solution II was added, mixed and made up to 50 mL with water. A drop of ethanol was added to suppress the foam. The mixture was then filtered using filter paper and the first 10 mL of filtrate was discarded. Five millilitres of the mixture were pipetted, each into two test tubes. Five millilitres of water were added to the first test tube—identified as the sample solution; 5 mL of sodium bisulphite solution (0.2%) was added to the second test tube—identified as the reference solution. The absorbance reading of sample solution against the reference solution was determined using spectrophotometer at 284 nm and 336 nm in quartz cell within 1 h after sample preparation. Should the absorbance exceed 0.6 at 284 nm, the sample solution would be diluted with water, while the reference solution would be diluted with sodium bisulphite in the same order. The calculation of HMF content was in mg/kg:


(2) $$\text{HMF in mg}/\text{kg}=({\text{A}}_{284}-{\text{A}}_{336})\times 149.7\times 5\times \text{D}/\text{W}$$

where A_284_ = absorbance at 284 nm, A_336_ = absorbance at 336 nm, D = dilution factor if dilution is necessary, and W = weight of the honey in g.

### Proline Content

Honey was diluted with distilled water to produce a 5% (w/v) solution, and 0.5 mL of the solution was mixed with 0.025 mL of formic acid. Then, 1 mL of ninhydrin was added into the mixture before being placed in boiling water for 15 min. The mixture was let to cool for 5 min at 22°C before added with 5 mL of isopropanol. The absorbance was read using spectrophotometer at 520 nm against a blank, and a calibration curve was generated using solution of standard of known concentrations.

### Total Phenolic Content (TPC)

The TPC in honey samples were quantified using a modified spectrophotometric method (Singleton *et al*. 1999). The sample was prepared by mixing 1 mL of honey with 1 mL of Folin-Ciocalteu reagent. After 3 min, 1 mL of 10% sodium carbonate (w/v) solution was added to the mixture and 3 mL of distilled water was added later. The mixture was kept in the dark for 90 min to allow for oxidation-reduction reaction to take place before absorbance reading was taken. The absorbance was read using spectrophotometer. Gallic acid was used to generate a standard curve, which would be used to determine the TPC and expressed as mg/kg of gallic acid equivalents (GAEs) of honey.

### Reducing Sugar Content (RSC)

Honey (0.1 g/mL) was diluted by 100-fold with ultra-pure water and shaken to mix. Then, 1 mL aliquot of diluted honey solution was mixed with 1 mL of 3-amino-5-nitrosalicylic acid solution and incubated in a boiling water bath (DAIHAN Scientific Co. Ltd., South Korea) for 10 min. Next, the mixture was allowed to cool down and 7.5 mL of ultra-pure water was then added into the mixture. The absorbance was measured at 540 nm using spectrophotometer (Saxena *et al*. 2010). Glucose solution was used to generate a standard curve to determine RSC, which was expressed as g/100 g honey.

### Protein Content

Protein content in the honey was determined using Lowry Assay. The sample was prepared by mixing 1 mL of honey with 1 mL of ultra-pure water. Then, 4 mL of Biuret reagent was added into the mixture and incubated for 10 min. Next, 0.5 mL Folin-Ciocalteu reagent was added into the mixture and incubated in the dark for 30 min. The absorbance was measured at 660 nm using spectrophotometer. A Bovine Serum Albumin (BSA) solution of known concentrations was used to generate a standard curve for protein content determination.

### Ash Content

Honey samples of 5 g each were placed in porcelain crucibles and heated for 24 h in the oven (Binder FD 115/E2, Germany) at 100°C, followed by calcination in muffle oven at 550°C. The samples were weighed following the incubation and the data were recorded.

### Mineral Content

Honey samples of 5 g each were placed in porcelain crucibles and heated at 100°C in an oven (Binder FD 115/E2, Germany) for 24 h, followed by calcination at 550°C in muffle oven. The ashes were dissolved in 50 mL of 5% nitric acid and stirred. The solutions were filtered using a syringe filter with an outer ring of a size up to 25 mm and a hydrophilic PTFE membrane of 0.45 μm pore size; the filtrates were analysed using inductively coupled plasma optical emission spectroscopy (ICP-OES; PerkinElmer, USA). Concentration of metal elements in the filtrates were quantified based on the standard curve generated using standard solution of known metal contents (0.1 ppm, 0.5 ppm and 1.0 ppm); the analysis was conducted concurrently. The calcium (Ca), copper (Cu), iron (Fe), magnesium (Mg), sodium (Na), potassium (K) and zinc (Zn) concentrations were determined at 317.9 nm, 327.4 nm, 238.2 nm, 285.2 nm, 589.6 nm, 766.5 nm and 206.2 nm, respectively. The honey samples were prepared and tested for metal contents in triplicate for data quality assurance.

### Statistical Analysis

The data were tested for normality before further analysis. Analysis of variance (ANOVA) and Kruskal-Wallis H test (for non-parametric variables) were used to test the level of significance in the differences between honeys of different sources. To determine the possible association between measured variables, unsupervised multivariate statistical technique, i.e., principal component analysis (PCA) was performed. Statistical analysis was conducted using R (version 4.0.3) and IBM SPSS Statistics for Windows version 22.0.

## RESULTS

A total of 76 honey samples were examined in this study: 25 samples from contract beekeepers rearing *A. cerana*; 17 samples of unknown sources from different vendors; and 34 samples of various brands from supermarkets in Sabah. Table 1 shows the physicochemical properties and mineral contents of the honey sampled in this study and the comparison between the sources.

### pH

There was a significant difference (H = 30.47; *p* < 0.01) between honey from CB and honey from UNS and BH. According to the results, honey from UNS was the most acidic (pH 2.9 ± 0.26), but not significantly different from that of BH.

### Electrical Conductivity (EC)

The results showed that EC of honey from CB was significantly higher (H = 35.36; *p* < 0.01), about twice as high as UNS and BH (0.781 ± 0.214, 0.389 ± .082 and 0.356 ± 0.232 mS/cm, respectively).

### Colour (Pfund)

The colour of the honeys examined in this study ranged from white (low Pfund) to dark amber (high Pfund), with the colour of the honey from CB being darker than that of UNS and BH. This study showed a significant difference (H = 25.42; *p* < 0.01) in Pfund between honey from CB (101.1 ± 36.1) and honey from UNS and BH (52.6 ± 23.3 and 60.3 ± 28.9 Pfund, respectively).

### Colour Intensity (mAU)

There was a significant difference in the colour intensity of honey from different sources (F = 42.4; *p* < 0.01), with honey from CB having a higher intensity (407.1 ± 239.4) than that from UNS and BH (303.6 ± 150.7 and 351.8 ± 394.6 mAU, respectively).

### Total Sugar Content (TSC)

The TSC value of honey from CB (74.2 ± 2.49 g/100 g honey) was significantly (F = 21.31; *p* < 0.001) lower than that of UNS (78.0 ± 3.3 g/100 g honey) and BH (79.7 ± 1.7 g/100 g honey).

### Hydroxymethylfurfural (HMF) Content

Honey from UNS had the lowest HMF content (81.5 ± 78.1 mg/kg), which was significantly different (H = 22.06; *p* < 0.001) than honeys from CB (182.6 ± 57.2 mg/kg) and BH (196.7 ± 81.2 mg/kg).

### Proline Content

The honey from UNS had the lowest proline content (0.66 ± 1.45 mg/kg), which was significantly different (H = 27.53; *p* < 0.01) from the honey samples from CB (5.55 ± 2.67 mg/kg) and BH (12.0 ± 12.8 mg/kg).

### Total Phenolic Content (TPC)

There was no significant difference in TPC between the honeys from the different sources examined in this study (H = 5.22; *p* > 0.05). Honey from CB contained the highest phenolic content, followed by BH and UNS (4639 ± 1452, 4312 ± 2131 and 3673 ± 1132 mg/kg GAE, respectively).

### Reducing Sugar Content (RSC)

There was no significant difference in the RSC of honey from different sources studied (H = 3.45; *p* > 0.05). The RSC of honey from UNS was the highest, followed by honeys from CB and BH (10.42 ± 4.8, 9.4 ± 2.5 and 8.5 ± 3.65 g/100 g, respectively).

### Protein Content

This study also found no significant difference in the protein content of honey from different sources (H = 0.48; *p* > 0.05). Honey from UNS contained the highest protein content, followed by honey from CB and BH (0.26 ± 0.01, 0.25 ± 0.01 and 0.24 ± 0.03 g/kg, respectively).

### Ash Content

Honey from BH had the highest ash content compared to honey from CB and UNS (0.74 ± 1.85, 0.54 ± 0.93 and 0.28 ± 0.29 g/100 g, respectively), but no significant difference was found between these honeys (H = 1.92; *p* > 0.05).

### Mineral Content

Table 2 shows a statistically significant difference (*p* < 0.05) in the mineral content (Ca, Cu, K, Na, Mg and Zn) of honeys from different sources. The mineral content of honey from CB was up to four times higher than that of UNS and BH for some minerals.

### Correlation between Parameters

Table 3 shows the Spearman’s correlation coefficients for the different properties of honey determined in this study. Ash content, pH and EC showed positive and significant correlation (ρ > 0.3, *p* ≤ 0.01) with all the elements present in the honey. The K content and colour (mm Pfund) of the honey had the most significant correlation with the measured properties (16 parameters each), while HMF and RSC had the lowest correlation with the honey properties reported in this study (six parameters each). Element K was the main component of ash that showed the highest correlation (ρ = 0.408, *p* ≤ 0.01) compared to other elements (Kek, *et al*. 2017a). However, the freshness of honey as represented by HMF content cannot be determined by colour (Pfund) as the relationship was found to be insignificant with a positive correlation (ρ = 0.215, *p* > 0.05). Colour intensity and colour (Pfund) have a positive significant correlation with TPC (ρ = 0.796, *p* ≤ 0.01 and ρ = 0.643, *p* ≤ 0.01, respectively). On the other hand, TSC in honey was negatively correlated with all minerals, although almost all minerals showed significant relationships with TSC. In contrast, RSC in the honeys examined in this study showed a weak, non-significant correlation with mineral content, suggesting that the presence of sugar has no relationship with mineral content.

### Principal Component Analysis

**Figure 1:**
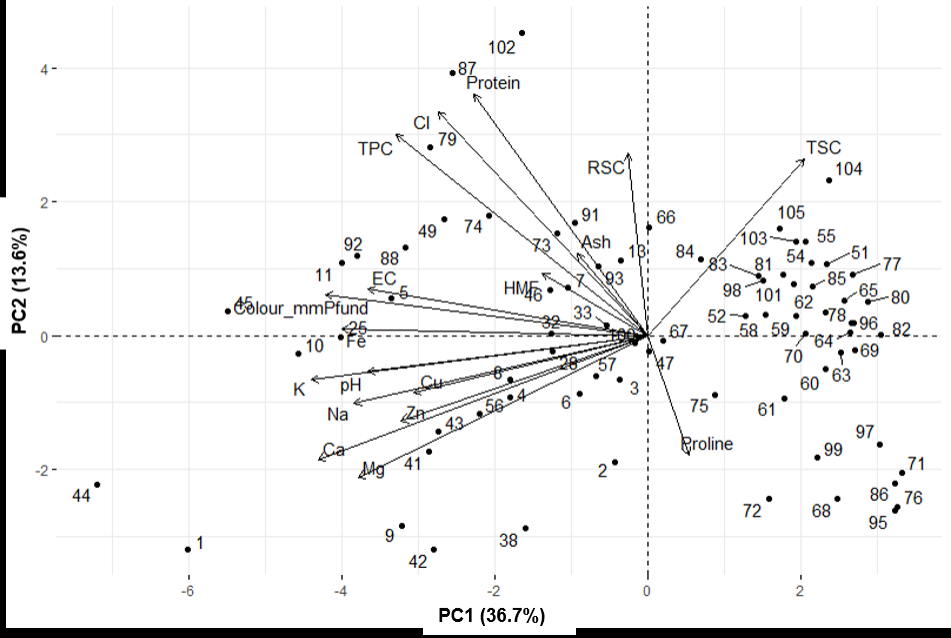
PCA for the honey variables.

Principal component analysis (PCA) was performed to analyse the similarities between honeys from different sources. Five components with eigenvalues greater than 1 were extracted and these components explained 72.25% of the data variation. Only the first two components were considered compelling for explanations, as the scree plot shows a straight line after the second principal component (PC) (Fig. 1). PC1 explained 36.7% of the data variability with a total eigenvalue of 6.60, while PC2 explained 13.6% of the data variability with a total eigenvalue of 2.45. Based on PCA, the negative contributions to PC1 were pH (7.09%), EC (7.1%), Pfund (8.17%), TPC (6.37), Ca (8.35%), Cu (5.93%), Fe (7.75%), K (8.52%), Mg (7.34%), Na (7.43%) and Zn (6.24%). Meanwhile, CI (11.55%) and protein content (12.5%) contributed positively to PC2.

**Figure 2:**
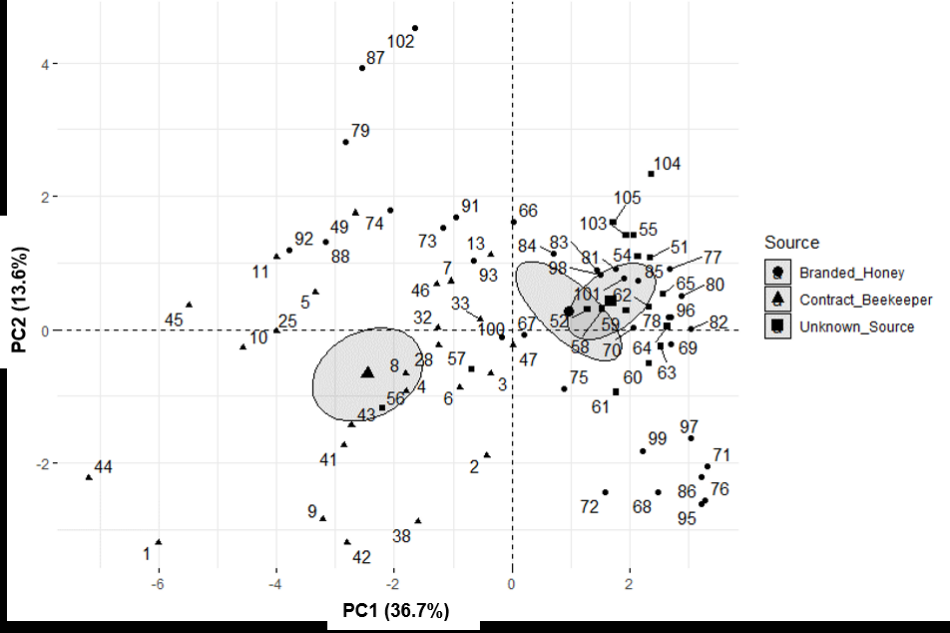
PCA for the sources of honey.

Fig. 2 shows a clear separation of honeys from different sources (CB, UNS and BH) in Sabah. The honeys from UNS and BH are at the positive values and overlap, indicating a high similarity between these two sources. None of the honeys from CB are at the positive values of PC1, especially for mineral content. Analysis of variance for PC1 showed a significant difference between honeys from CB and honeys from UNS and BH (F = 13.46, *p* < 0.05). Similarly, for PC2, ANOVA also showed a significant difference between honey from CB and honey from UNS and BH (F = 8.52, *p* < 0.05).

**Figure 3:**
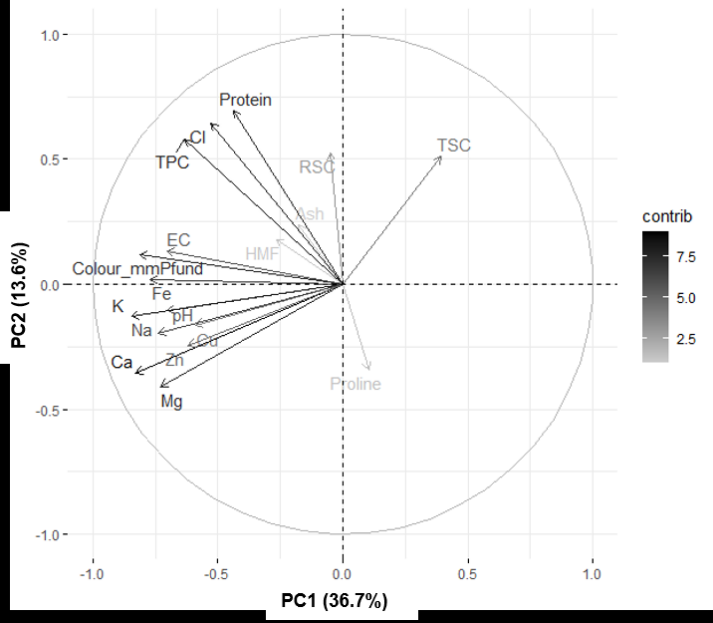
Loading plot for PC1 vs. PC2 based on measured variables.

Fig. 3 shows the loading plot of PC1 and PC2, a tool to observe the correlation between the variables and PC. All mineral contents (Ca, Cu, Fe, K, Mg, Na and Zn) play a significant role in determining PC1 in this study. The honey of CB is significantly different from that of UNS and BH and has higher mineral content as shown in Table 2.

## DISCUSSION

In general, honeys from UNS and BH are more acidic (pH 3.22–4.03) compared to most honeys produced in Malaysia (Moniruzzaman *et al*. 2013; Kek, Chin, Tan, *et al*. 2017), which is below the established pH limit (pH 3.4–6.1). On the other hand, honey from CB is comparable to honey from forest (Chua *et al*. 2012) and other monofloral honeys (Moniruzzaman *et al*. 2013; Kek, Chin, Yusof, *et al*. 2017). The floral sources and sugar fermentation affect the acidity of the honey as sugar is converted to alcohol. The conversion of glucose in honey to gluconic acid by glucose oxidase contributes to the low pH of honey. The presence of bioactive compounds in honey, such as those of the phenolic group, also lowers the pH (Pasupuleti *et al*. 2017) while contributing to the flavour and antimicrobial properties (Bogdanov *et al*. 2008).

The total value of EC for honey obtained in this study is within the range of EC reported for honey produced in Peninsular Malaysia (0.35–1.08 mS/cm; see Moniruzzaman *et al*. 2013 and Kek, Chin, Yusof, *et al*. 2017). The EC of honey from CB, sourced from a polyfloral environment, is comparable to EC of monofloral honey sourced from meadow and sunflower fields in Serbia (Sakač *et al*. 2019) and from acacia and tualang honey in Malaysia (Moniruzzaman *et al*. 2013; Kek, Chin, Yusof, *et al*. 2017), but lower than the recommended Codex Alimentarius (FAO & WHO 2019) value. On the other hand, the EC of honey from CB is higher than that of stingless bees, as in Brazil (*Melipona* spp.; 0.15–0.66 mS/cm; see Ávila *et al*. 2019) and China (*Lepidotrigona flavibasis*; 0.54 mS/cm; see Wu *et al*. 2020); however, the value is lower than that of *Heterotrigona itama* (1.08 mS/cm) as reported in Peninsular Malaysia (Kek, Chin, Yusof, *et al*. 2017). The EC of honey is generally influenced by the floral source, mineral salts, acidity, ash content and viscosity of the honey. Previously, EC has been suggested as an indicator for determining authenticity, entomological species, botanical and geographical origin of honey (Kek, Chin, Yusof, *et al*. 2017; Sakač *et al*. 2019) and the results of this study show that high EC can potentially be used as an indicator for distinguishing honey sources. The honeys collected in this study generally have a lower Pfund than honeys in Ireland (Kenyan, blended, monofloral, rural and urban) (Kavanagh *et al*. 2019) and Spain (chestnut honey and honeydew honey) (Rodríguez-Flores *et al*. 2019), but a higher Pfund than that of stingless bee honeys (Ávila *et al*. 2019). The colour of honey from CB is comparable to that of Saudi Arabia, while the honeys from UNS and BH are comparable to those of Yemen and Egypt (El Sohaimy *et al*. 2015). The colour of honey is attributed to its origin, which may be an important quality criterion for honey. For example, blossom honey is generally lighter in colour than honeydew honey (Pita-Calvo & Vázquez 2017), and even among monofloral honey can vary considerably in colour (Ferreira *et al*. 2009). Light-coloured honey generally has lower TPC but higher DPPH scavenging activity, reducing power and β-carotene bleaching inhibition compared to darker honey (Ferreira *et al*. 2009).

The CI of honeys determined in this study is within the range (320.3–580.7 mAU) reported for honeys of most honey bee species found in Peninsular Malaysia, but lower than that of stingless bees (512–1141.3 mAU) and Manuka honey (7296.7 mAU) (Moniruzzaman *et al*. 2013; Kek, Chin, Yusof, *et al*. 2017; Selvaraju *et al*. 2019). Similar to Pfund, colour intensity has often been used as an indicator of honey quality, e.g., the degree of antioxidant activities by biological pigments (flavonoids, carotenoids, etc.). The higher the colour intensity, the higher the antioxidant activity of the honey, as demonstrated by several authors (Cimpoiu *et al*. 2013; Moniruzzaman *et al*. 2013; Kek, Chin, Yusof, *et al*. 2017).

In general, the TSC of honeys from *A. cerana* determined in this study are also higher than the TSC of local monofloral honey as reported by Moniruzzaman *et al*. (2013) and Selvaraju *et al*. (2019), and those of stingless bee species (Ávila *et al*. 2019; Shamsudin *et al*. 2019; Wu *et al*. 2020). This study shows that the TSC of honey in Sabah is comparable to the TSC of honey in Peninsular Malaysia (65.53–81.93 g/100 g honey; see Moniruzzaman *et al*. 2013 and Kek, Chin, Tan, *et al*. 2017). The TSC value measures the solid-soluble content of all sugars present in honey, which includes nectar from plants or liquid excreted by other insects. The TSC value is also influenced by the origin of foraging sources and foraging preferences (Lan *et al*. 2021), which has a crucial impact on the maturity of the honey (Ma *et al*. 2019). It is worth noting that the TSC value can potentially be an important indicator for determining honey quality. In general, blossom honey has a significantly higher TSC value than honeydew honey (Manzanares *et al*. 2011). Wolff (2006) observed a weak but significant correlation between floral visitation and nectar sugar composition. The results show that honey in Sabah is generally high in HMF compared to honeys investigated in previous studies in Malaysia (0.26–68.99 mg/kg) (Moniruzzaman *et al*. 2013; Kek, Chin, Tan, *et al*. 2017) and Serbia (Sakač *et al*. 2019). The level is also above the range set by the Malaysia Food Act 1983 (Food Act 1983 (Act 281) & Regulations 2019) and Codex Alimentarius (FAO & WHO 2019). The HMF content is an indicator of the freshness of the honey, which is influenced by the age of the honey and the processing method used (Önür *et al*. 2018). Prolonged thermal treatment can break down sugars and lead to a Maillard reaction and thus to the formation of HMF. In addition, the type of storage can also influence the HMF content in honey, whether in natural hives or under other storage conditions such as unstable ambient temperature (Khalil *et al*. 2010; da Silva *et al*. 2016). For example, high HMF content was found in Tualang honey stored for a long period of time, from 2.80–24.87 mg/kg (3–6 months) to 128.19–1,131.76 mg/kg (12–24 months). The presence of invert sugar syrup in adulterated honey also contributes to the formation of HMF, as honey is used as a food additive. The high HMF value in this study is due to the long sampling, transport and storage leading to the formation of HMF.

The proline content in honey from CB is lower than that of some monofloral and most polyfloral honeys in Malaysia, Spain, Italy and Poland (Moniruzzaman *et al*. 2013; Chua & Adnan 2014; Seraglio *et al*. 2019). According to a previous study, some Malaysian monofloral honeys (acacia, rubber and oil palm) have proline content ranging from 0.002 to 16.35 mg/kg, while the value for polyfloral honey is up to 628.69 mg/kg; the minimum recommended value for pure honey is 180 mg/kg, depending on the type of honey (Bogdanov *et al*. 1999). Proline is an amino acid that accounts for 50%–85% of the total amino acids in honey and is formed when bees convert nectar into honey. Proline content is used as an indicator to determine the maturity and authenticity of honey, which also reflects the botanical and geographical origin of the honey (Cotte *et al*. 2004; Wen *et al*. 2017). However, non-adulterated honey can also have a proline content as low as 0.002 mg/kg (Chua & Adnan 2014); the highest value can be as high as 9,600 mg/kg (Seraglio *et al*. 2019).

The TPC of the honeys reported in this study is generally higher than that of other honeys produced by honey bees and stingless bees in Peninsular Malaysia (Moniruzzaman *et al*. 2013; Selvaraju *et al*. 2019; Shamsudin *et al*. 2019). Nutrients are transported through the phloem to all parts of the plant, either in the form of beneficial or toxic compounds. Zhang *et al*. (2016) reported that phenolic compounds in nectar act as attractants for honey bees. Therefore, TPC has been proposed as an indicator for determining the authenticity of honey and as a marker for profile characterisation that can be used for honey quality assessment and classification (Cheung *et al*. 2019; Vasić *et al*. 2019).

The RSC value of the honeys tested in this study was lower than that of honeys produced in Peninsular Malaysia (40–92 g/100 g) (Moniruzzaman *et al*. 2013; Selvaraju *et al*. 2019), which is below the range specified by the Malaysia Food Act 1983 (Food Act 1983 (Act 281) & Regulations 2019). Reducing sugars consist of monosaccharides (fructose, galactose and glucose) and in some cases disaccharides (maltose). The presence of RSC in honey is crucial for predicting crystallization (Escuredo *et al*. 2014), as exceeding the saturation level leads to the formation of crystals in honey (Bhandari & Bareyre 2003), which affects its shelf life (Elamine *et al*. 2020).

The protein content of the honeys reported in this study was lower than that of honeys produced in Peninsular Malaysia (0.6–10 g/kg) (Moniruzzaman *et al*. 2013; Chua & Adnan 2014; Kek, Chin, Tan, *et al*. 2017). The protein content in honey comes from external sources generated by foraging activity, as well as salivary enzymes and the hypopharyngeal gland of honey bees. The total protein content in honey influences the maturity level of the honey (Ma *et al*. 2019).

The average ash content of the honeys examined in this study was less than 1%, which is the recommended level under the Malaysia Food Act 1983 (Food Act 1983 (Act 281) & Regulations 2019). However, lower ash content was found in monofloral honeys from Malaysia (0.05–0.19 g/100 g) (Chua & Adnan 2014; Kek, Chin, Tan, *et al*. 2017), Serbia (0.08–0.15 g/100 g) (Sakač *et al*. 2019) and Romania (0.09–0.4 g/100 g) (Al *et al*. 2009) than in the honeys in this study. The ash content of the polyfloral honeys reported in this study was also higher than that of other locally produced honeys (0.23–0.27 g/100 g) (Chua & Adnan 2014; Kek, Chin, Tan, *et al*. 2017) and honeys produced in Kashmir (0.3 g/100 g) and Saudi Arabia (0.23 g/100 g) (El Sohaimy *et al*. 2015). This study also reports higher ash content than in honeys produced by stingless bees in Malaysia (Shamsudin *et al*. 2019) and Brazil (Ávila *et al*. 2019). The ash content in honey consists mainly of inorganic compounds such as Ca, Fe, K, Mg and Na. These minerals can come from a botanical source, external contaminants (processing and storage) and environmental pollution (dust and smoke). Ash content influences the EC of the honey, which is often used as an indicator of the botanical and geographical origin of the honey.

Several studies in Malaysia have found higher mineral content in forest honey than in honey from other sources (Chua *et al*. 2012; Moniruzzaman *et al*. 2014; Kek, Chin, Tan, *et al*. 2017; Cheng *et al*. 2019). Only a fraction of the samples from BH noted the rearing site in the forest, while the rest remain hidden. Although the samples from UNS are believed to be from the forest, it is difficult to identify the source given the place of sale due to uncooperative sellers.

Element K was the most abundant mineral in this study (2.65 ± 1.44 g/kg), followed by Ca, Na, Mg, Fe, Cu and Zn. This element had the highest value in honeys from BH and UNS (276.3 ± 657.9 mg/kg and 276.3 ± 657.9 mg/kg, respectively). The element was also reported as the most abundant mineral in the honeys studied in other studies conducted in Malaysia (95.4–4,026.4 mg/kg) (Chua *et al*. 2012; Moniruzzaman *et al*. 2014; Kek, Chin, Tan, *et al*. 2017) and elsewhere in the world (see Solayman *et al*. 2016; Jovetić *et al*. 2017; Sakač *et al*. 2019; Wu *et al*. 2020). In general, K appears to be the most abundant mineral in all classes of honey produced by honey bees or stingless bees (Solayman *et al*. 2016; Kek, Chin, Tan, *et al*. 2017; Cheng *et al*. 2019).

The element Ca was the second most abundant mineral in the honeys from CB and UNS, but the third highest in honey from BH (458.4 ± 205, 143.3 ± 61.9 and 145.5 ± 92.1 mg/kg, respectively). This study also found the highest Na content in honey compared to honeys investigated in previous studies in Malaysia (Chua *et al*. 2012; Kek, Chin, Tan, *et al*. 2017). In addition, the mineral contents, namely K, Ca, and Na, are higher in honey from CB compared to Manuka honey (Moniruzzaman *et al*. 2014) and other monofloral honeys (acacia, linden, sunflower, rapeseed and basil) (Jovetić *et al*. 2017). Elements such as K, Ca and Mg are essential for plant growth and are therefore called macronutrients. The element K generally acts as an activator for enzymes and facilitates osmoregulation. The element Ca is crucial for regulating enzyme activities, cell division and adhesion, while Mg is an integral part of the chlorophyll molecule and also acts as a regulator in enzyme reactions. It is therefore not surprising that K is abundant in plants.

Unlike other macronutrients, Zn was the least abundant mineral in the honey of CB, UNS and BH (3.17 ± 2.73 mg/kg, 1.46 ± 1.27 mg/kg and 1.48 ± 0.95 mg/kg, respectively). The Zn content in honey from CB was comparable to that of Tualang honey (3.316 ± 0.619 mg/kg) and Gelam (3.045 ± 0.003 mg/kg) found in Malaysia (Chua *et al*. 2012). In plants, Zn is considered a micronutrient that plays a crucial role in stabilising RNA and DNA structures and also acts as an activator for some enzymes involved in plant defence against pathogens. All these nutrients are naturally present in tropical soils and can be taken up by plants.

This study also shows that colour can determine honey quality (CI and Pfund), which has a significant positive correlation with all parameters (except TSC, which has a negative correlation), similar to previously reported results (Kavanagh *et al*. 2019). This relationship suggests that honey colour is closely related to phenolic content: the darker the colour, the higher the TPC, which is confirmed by previous studies (Moniruzzaman *et al*. 2013; Kek, Chin, Yusof, *et al*. 2017).

This result suggests that the quality of honey varies according to its sources, and that a clear separation according to the sources of the honey can be achieved by profiling the mineral content, which in turn can be used as an indicator of the origin of the honey. The loading diagram in this study shows slight differences in the grouping of minerals. While all minerals in this study are grouped in a similar PC, Jovetić *et al*. (2017) reported that only K, Ca and Mg are grouped and classified as an important PC2. The tropical forest is rich in minerals, with high plant diversity leading to a wide range of honey properties. Honey colour and TPC have a positive effect on PC2 and contribute significantly to this component. Honey colour has been shown to correlate significantly with TPC in honey in this and other studies (Moniruzzaman *et al*. 2013; Moloudian *et al*. 2018), with darker honey having higher phenolic content. In contrast, other parameters such as ash content, HMF and RSC contributed least to explaining the factor.

## CONCLUSION

This study is the first to report the physicochemical properties and mineral content of raw honey from three primary honey sources: Contract beekeepers, street vendors of unknown honey sources, and branded honey sold in local supermarkets in Sabah. The data show that honey from these sources differs significantly in terms of physicochemical properties and mineral content. Honey from contract beekeepers who have reared their bees in the forest has been shown to be of better quality as it has a higher mineral content. The results suggest that honey from bees reared in the forest could be a good choice when consumers or the food sector consider honey with high nutrient and mineral content.

## Figures and Tables

**Table 1:** Comparison in the properties of honey available in Sabah.

Properties	Contract beekeeper	Unknown source	Branded honey
pH	3.42 ± 0.13^a^	2.90 ± 0.26^b^	3.10 ± 0.32^b^
EC, (mS/cm)	0.781 ± 0.214^a^	0.389 ± 0.082^b^	0.356 ± 0.232^b^
Colour intensity (mAU)	407.1 ± 239.4^a^	303.6 ± 150.7^b^	351.8 ± 394.6^ab^
Colour (mm Pfund)	101.1 ± 36.1^a^	52.6 ± 23.3^b^	60.3 ± 28.9^b^
TSC (g/100 g)	74.2 ± 2.49^a^	78.0 ± 3.3^b^	79.7 ±1.7^b^
HMF content (mg/kg)	182.6 ± 57.2^a^	81.5 ± 78.1^b^	196.7 ± 81.2^a^
Proline content, (mg/kg)	5.55 ± 2.67^a^	0.66 ± 1.45^b^	12.0 ± 12.8^a^
TPC (mg/kg GAE)	4639 ± 1452	3673 ± 1132	4312 ± 2131
RSC (g/100 g)	94.7 ± 25.7	104.2 ± 48.2	85.3 ± 36.5
Protein content, (g/kg)	0.254 ± 0.01	0.255 ± 0.01	0.243 ± 0.03
Ash (g/100 g)	27.2 ± 46.3	13.9 ± 14.6	37.1 ± 92.7

*Notes:* Mean ± standard deviation. Values with at least one similar alphabet in the same row are not significantly different (*p* > 0.05).

**Table 2:** Comparison of mineral elements in honey.

Mineral, mg/kg	Contract beekeeper	Unknown source	Branded honey
Ca	458.4 ± 205^a^	143.3 ± 61.9^b^	145.5 ± 92.1^b^
Cu	5.82 ± 4.42^a^	2.92 ± 2.98^b^	3.34 ± 4.31^b^
Fe	8.24 ± 5.88^a^	2.46 ± 1.23^b^	6.31 ± 5.18^b^
K	2,654.5 ± 1438.5^a^	276.3 ± 657.9^b^	629.3 ± 1,332.65^b^
Mg	166.0 ± 108.5^a^	57.5 ± 87.4^b^	43.5 ± 25.78^b^
Na	307.8 ± 179.9^a^	63.8 ± 34.1^b^	169.7 ± 172.3^b^
Zn	3.17 ± 2.73^a^	1.46 ± 1.27^b^	1.48 ± 0.95^b^

*Notes*: Mean ± standard deviation values. Alphabets with a different superscript in the same row are significantly different (*p* < 0.05)

**Table 3:** Spearman’s rank-order correlation coefficient between measured properties of honey.

	pH	EC	Ash	CI	Pfund	HMF	TPC	Proline	Protein	RSC	TSC	Ca	Cu	Fe	K	Mg	Na	Zn
pH	1.000																	
EC	0.514**	1.000																
Ash	0.238*	0.221	1.000															
CI	0.340**	0.529**	0.331**	1.000														
Pfund	0.730**	0.612**	0.393**	0.689**	1.000													
HMF	0.273*	0.151	0.031	0.231*	0.215	1.000												
TPC	0.437**	0.473**	0.366**	0.796**	0.643**	0.224	1.000											
Proline	0.394**	0.136	0.195	0.094	0.311**	0.187	0.245*	1.000										
Protein	0.159	0.306**	0.254*	0.583**	0.426**	0.261*	0.586**	−0.085	1.000									
RSC	0.142	0.245*	0.253*	0.376**	0.262*	0.048	0.228*	−0.248*	0.221	1.000								
TSC	−0.371**	−0.363**	0.121	0.093	−0.329**	−0.041	0.038	−0.009	0.089	0.196	1.000							
Ca	0.577**	0.668**	0.357**	0.381**	0.676**	0.156	0.395**	0.183	0.126	0.144	−0.510**	1.000						
Cu	0.461**	0.431**	0.358**	0.325**	0.653**	0.061	0.327**	0.271*	0.078	0.176	−0.268*	0.711**	1.000					
Fe	0.589**	0.444**	0.350**	0.369**	0.620**	0.259*	0.476**	0.446**	0.259*	0.087	−0.063	0.539**	0.526**	1.000				
K	0.674**	0.645**	0.408**	0.458**	0.781**	0.248*	0.548**	0.374**	0.302**	0.185	−0.382**	0.820**	0.672**	0.767**	1.000			
Mg	0.713**	0.617**	0.364**	0.382**	0.779**	0.131	0.409**	0.278*	0.116	0.147	−0.511**	0.925**	0.788**	0.592**	0.818**	1.000		
Na	0.610**	0.531**	0.339**	0.348**	0.631**	0.281*	0.439**	0.395**	0.135	0.165	−0.241*	0.770**	0.719**	0.776**	0.835**	0.797**	1.000	
Zn	0488**	0.390**	0.310**	0.440**	0.672**	0.157	0.411**	0.234*	0.192	0.213	−0.282*	0.563**	0.517**	0.658**	0.682**	0.652**	0.624**	1.000

*Notes:* EC = Electrical conductivity, CI = Colour intensity, HMF = Hydroxymethylfurfural, TPC = Total phenolic content, RSC = Reducing sugar content, TSC = Total sugar content, Ca = Calcium, Cu = Copper, Fe = Iron, K = Potassium, Mg = Magnesium, Na = Sodium, Zn = Zinc.

* *p* ≤ 0.05,

** *p* ≤ 0.01
